# Smokeless Tobacco and Dual Use among Firefighters in the Central United States

**DOI:** 10.1155/2013/675426

**Published:** 2013-03-06

**Authors:** Nattinee Jitnarin, Christopher K. Haddock, Walker S. C. Poston, Sara Jahnke

**Affiliations:** Center for Fire Rescue and EMS Health Research, Institute for Biobehavioral Health Research, National Development and Research Institutes, 1920 West 143rd Street, Leawood, KS 66224, USA

## Abstract

Little is known about smokeless tobacco (SLT) use in the fire service, whose personnel need to maintain high levels of health and fitness given the rigorous physical and mental job requirements. We examined the relationships among variables associated with SLT use and dual tobacco use (SLT and smoking) among 353 male career firefighters. Around 13% of male career firefighters reported being current exclusive SLT users, and 2.6% used both cigarettes and SLT. Age-adjusted models revealed that race, binge drinking, and dietary fat consumption were positively associated with exclusive SLT use when compared to nontobacco users. SLT users were much more likely to binge drink (OR = 3.98, *P* < .01) and consume high fat foods (OR = 1.94, *P* < .05). Only high dietary fat consumption was a strong correlate (OR = 8.41, *P* < .05) of dual use when compared to nontobacco users. SLT and dual tobacco use are associated with significant health risks. Detailed information on the predictors of SLT use among firefighters will aid in developing more effective tobacco prevention and cessation intervention in fire service.

## 1. Introduction

Smoking is the leading preventable cause of morbidity and mortality in the USA. In the last decades however, rates of cigarette smoking have decreased. In contrast, the use of the other forms such as smokeless tobacco (SLT) has increased [1, 2]. This suggests that smokers might use SLT as a substitute for cigarettes due to restrictions on smoking (e.g., indoor smoking laws), participation in smoking cessation programs, and/or the perception that SLT products confer lower morbidity and mortality risks [3]. In addition, various SLT products have been suggested as alternatives to smoking and/or as a harm reduction strategy [4], but there is limited evidence establishing the effectiveness of such an approach [5]. In contrast, some data suggest that SLT use significantly increases the subsequent use of cigarettes and dual use of cigarettes and SLT [6, 7]. Furthermore, a number of studies concluded that SLT use is related to several types of cancer (i.e., oral cancer, pancreatic cancer) [8].

Rates of SLT use are high among US military personnel relative to the general US population [9, 10]. However, little is known about SLT use in the fire service, which shares some cultural characteristics with the military and whose personnel also need to maintain high levels of health and fitness given the rigorous physical and mental job requirements [11]. Only two studies to date have reported tobacco use prevalence among firefighters. Lee and colleagues [12] estimated that the average cigarette smoking prevalence among firefighters from 1987 to 1994 was 26.9% (SD = 3.7), although this estimate was based on a small sample of firefighters, only reported cigarette use, and represented the average prevalence over a period nearly two decades ago. A recent study conducted by Haddock and team [13] found that the rates of firefighters' smoking and SLT use were 13.6% and 18.4%, respectively. Thus, while the rate of smoking was relatively low compared to the general US population, SLT use among firefighters was substantially higher than the populations they protect (i.e., a national unadjusted SLT use rate among adult males, 7.0% [14], a national SLT use prevalence among persons aged 12 and older, 3.5% [15]). The reduction in tobacco use over the last several decades and the subsequent rise in SLT use probably represent important policy and cultural changes in the fire service such as “no tobacco use” contracts as a condition of employment (where smoking is easy to monitor) and responses to indoor smoking and disease presumption laws [11]. 

Sociocultural and psychosocial variables that have been associated with an increase in SLT use include peer influence, gender, race, neighborhood environment, previous cigarette smoking, and alcohol use [16]. Several studies also found concurrent use of SLT and cigarettes among adults to be common [17–19]. This could raise the risk of nicotine exposure and tobacco-attributable morbidity and mortality among dual users when compared to those who use only cigarettes or SLT [19]. However, there currently are no studies assessing factors associated with SLT use or the characteristics of users and patterns of use in the fire service. The purpose of this study was to examine the relationships among variables associated with (1) SLT use and (2) dual tobacco use (SLT and smoking) among male career firefighters. 

## 2. Methods

### 2.1. Participating Fire Departments

The data reported are from the baseline evaluation of a longitudinal cohort study entitled “A prospective evaluation of health behavior risk for injury among firefighters” (EMW-2007-FP-02571). The primary aims of this study were to examine risk factors for injury in both career and volunteer firefighters in the International Association of Fire Chief's (IAFC) Missouri Valley region between May 2008 and May 2010. The protocol for the protection of human subjects for this study was approved by the National Development and Research Institutes, Inc., (NDRI) Institutional Review Board. 

 Sampling methodology and inclusionary criteria are presented in detail in previous reports [11, 13]. Briefly, firefighters from 11 randomly selected career and 13 randomly selected volunteer departments in the IAFC Missouri Valley Region comprised the sample for this study. A total of 736 firefighters who were available and on-duty during the baseline assessment were solicited and 714 (97%) agreed to participate and provided signed informed consent. Data from volunteer firefighters (*N* = 214) were not used because the focus of this study was on firefighter-related occupational exposures. In addition, data from 22 career firefighters who were either women or did not disclose their gender were excluded due to their low number and the resulting inability to examine the potential moderating impact of gender. Thus, a total of 478 career male firefighters had complete anthropometric data at baseline that comprised the initial sample for this study.

## 3. Measures

Tobacco use questions were modeled after national surveys such as the Behavioral Risk Factor Surveillance System [20] and the Department of Defense Survey of Health Related Behaviors among Active Duty Personnel [10]. Exclusive SLT users were those who reported current use of SLT products (chewing tobacco, dip, or snuff) and no current smoking. Dual users had smoked at least 100 cigarettes in their lifetime, had smoked a cigarette, even a puff, in the past 30 days, and reported current use of SLT products (chewing tobacco, dip, or snuff). Nontobacco use participants included those who reported never used any tobacco products and no current use of SLT and cigarettes. Firefighters who used cigars or cigarettes or any other forms of tobacco were excluded from the analysis. 

 Participants' body compositions were assessed. Height was measured by using a portable stadiometer. Body weight and body fat percentage (BF%) were determined with a digital scale and foot-to-foot bioelectrical impedance (Tanita 300, Tanita Corporation of America, Inc, Arlington Heights, IL). Waist circumference was determined by using a spring-loaded nonstretchable tape measure in accordance with recommendations from the US obesity guidelines [21].

 The Self-Report of Physical Activity (SRPA) questionnaire was used to estimate participants' physical activity levels. The SRPA provides a global, self-rating of physical activity patterns. Indicators of the questionnaire's validity in adult populations (i.e., significant correlations between SRPA ratings and measure maximal oxygen consumption (VO_2max⁡_)) have been established [22]. In this study, participants were asked to select a value from the questionnaire that best described their physical activity pattern during the past 30 days. These values, along with BMI, age, and gender, were used to estimate aerobic capacity (i.e., VO_2max⁡_). Aerobic capacity sufficient to exceed the National Fire Protection Association (NFPA) fitness standard was evaluated by comparing the estimated VO_2max⁡_ with the suggested cutpoint of ≥12 METS or ≥42 mL/kg/min) [23].

 Blood pressure was assessed using the Omron HEM-711AC (Omron Healthcare, Inc., Bannockburn, IL) in accordance with the Hypertension Detection and Follow-up program following the standard epidemiological protocol [24]. Participants were categorized as having elevated blood pressure if their systolic blood pressure was >120 mmHg and their diastolic pressure was >80 mmHg. 

 The Center for Epidemiological Studies Short Depression Scale (CES-D 10) was used to assess depressive symptoms [25]. The 10-item version was found to have comparable reliability estimates to those reported for the original CESD scale, and strong internal consistency (Cronbach's *α* = 0.92) and test-retest reliability (*r* = 0.83) [25]. The Perceived Stress Scale (PSS) is a 10-item measure that queries how unpredictable, overloaded, and uncontrollable individuals perceive their lives. The PSS has been found to be highly reliable in the general US population [26]. 

 Alcohol use was assessed by asking participants whether they had at least one drink of any alcoholic beverage in the past 30 days. Binge drinking was assessed by asking the participants to report the number of times during the past 30 days that they had five or more drinks of any alcoholic beverage on one occasion. Driving while intoxicated was determined by asking participants whether they drove a car or other vehicles on any occasion when they had too much to drink in the past 30 days. Items assessing alcohol use were modeled after the National Household Survey on Drug Use and Health [27] and the survey of military members [28]. In addition, the CAGE questionnaire was used to assess potential alcohol abuse [29]. Participants' affirmative responses were summed; scores equal ≥2 are considered to indicate potential problem drinking. 

 The Block Food Screener was employed to estimate fat and fruit/vegetable intake. The Block Food Screener is comprised of two primary sections; the meat/snacks and fruit/vegetable intake composite measures. It was designed to screen dietary fat and fruit and vegetable intake in the American diet [30]. Participants with scores <11 on fruit/vegetable screener were defined as low fruit/vegetable intake. High-fat intake was defined as having a meats/snacks screener score more than 22. The food screener has been found to be highly correlated (*r* = 0.60–0.70) with total fat, saturated fat, cholesterol, and fruit/vegetable intake when compared with the 1995 Block 100-item Food Frequency Questionnaire data [30]. 

Firefighter-specific health-risk behavior, that is, self-contained breathing apparatus (SCBA) use, was assessed by asking participants how often they used their SCBA during a fire and/or during salvage and overhaul.

## 4. Statistical Analyses

All statistical analyses were conducted using IBM SPSS 18.0 (SPSS, Chicago, IL). Odds ratios (ORs) were computed as measures of association for sociodemographics and health risk behaviors comparing current SLT users and dual users with nontobacco users as the referent group using binary logistic regression models with the backward elimination procedure. Predictors and prevalence of smoking and SLT prevalence in this cohort are reported in Haddock et al. [13]. 

## 5. Results

Complete data were available to classify 425/478 (89%) of the baseline participants into tobacco use categories. Of these baseline participants, 353 firefighters were included in the current data analysis; 13.3% (*n* = 57) were SLT users only, 2.6% (*n* = 11) were dual users of cigarettes and SLT, and 67.1% (*n* = 285) were nontobacco users. Descriptive data on characteristics of the study population, stratified by SLT use, are provided in Table 1.

SLT users were predominantly Caucasian with a college education or less, and a household income less than $75,000. Dual use was more prevalent among younger Caucasian participants who were less likely to have a college education and had family incomes of lower than $75,000. Less than half of nontobacco and SLT users had aerobic capacity sufficient to meet or exceed the suggested NFPA fitness criterion [23]. Compared to never users, SLT users and dual users reported higher rates of binge drinking and high-fat consumption. Interestingly, dual users were more likely to report driving under the influence during the past 30 days than their counterparts who never used tobacco. 

 Of participants who reported exclusive SLT use, the mean age at initiation was approximately 17 years old (Table 2), with most starting SLT use before joining the fire service (86.8%). On average, SLT users consumed almost 3.0 tins per week and around 4.9 (SD = 3.3) dips per day. Nearly half of current SLT users used SLT while on duty, and only 18% reported never using SLT during duty days. Among dual users, the average age of first SLT use was when they were 19 years old, compared with 15 years of age for cigarette initiation. In addition, the mean number of SLT and cigarettes used per day was 2.7 (SD = 2.9) dips and 6.6 cigarettes (SD = 7.4), respectively. Similar to SLT users, more than half of dual users already were using SLT before joining the fire service. There was no statistically significant difference in age of SLT initiation or level of SLT use between SLT users versus dual users. 

Table 3 presents the results of logistic regression models comparing nontobacco users with exclusive SLT users and dual users in relation to sociodemographic factors and health-risk behaviors. After age adjustment, race, binge drinking, and dietary fat consumption were found to be positively associated with exclusive SLT use. SLT users were more likely to binge drink (OR = 4.0, 95% CI = 1.7–9.3) and consume high fat foods (OR = 1.9, 95% CI = 1.0–3.6) when compared to nontobacco users. Only high dietary fat consumption was strongly associated (OR = 8.4, 95% CI = 1.1–67.2) with dual use status when compared to nontobacco users. 

## 6. Discussion

This study is the first to examine the characteristics of career fire service personnel who were classified as SLT users only or dual users of SLT and cigarettes, and factors associated with their use of these tobacco products. In our cohort of firefighters, the prevalence of exclusive SLT use was almost two times that found in national surveys of men, 13.4% versus 7.0% [2, 13], and was higher than rates from states represented in this study (i.e., current rates range from 7.2% in Colorado to 11.9% in South Dakota). In addition, the prevalence of dual use in our sample was 2.6%, which is 3-4 times higher than that found nationally, 0.6–0.8% [19]. 

Health behaviors associated with SLT or dual tobacco included alcohol intake and meat/snack consumption. Both SLT and dual users were almost 4 and 5 times more likely to binge drink than nontobacco users. Interestingly, dual users reported consuming meat and/or high-fat foods eight times more than nontobacco users. Dual users also were more involved in a number of health risk behaviors including reporting driving while intoxicated in the past 30 days, being less likely to eat fruit and vegetables, more likely to eat meat and high-fat foods, and less likely to use SCBAs during a fire. Although these risk behaviors of dual users were not found significant due to the low sample size, they were relatively distinct from those of both nontobacco and SLT users, indicating a unique group of tobacco users. 

Several study limitations should be noted. First, the sample consisted of career firefighters in one IAFC geographic region in the USA. Data may not be generalizable to other populations or regions. Second, tobacco use (except for cigarette smoking) was based on self-reports and not validated by biochemical markers, which could underestimate actual tobacco use. However, self-reports have been found to be valid for assessing all forms of tobacco use in several epidemiological studies [31], and self-reports of smoking were very accurate when compared to biochemically verification in this cohort [13]. Next, only 11 participants were dual users in this sample, which could influence and reduce the precision of the study outcomes. Finally, the study was cross-sectional, so drawing causal and directional inferences between the various forms of tobacco use and their correlates is not possible. 

Our findings have several public health implications. First, the trend toward clustering of tobacco use and other health-risk behaviors emphasizes the need to address these behaviors by using intervention strategies that consider multiple risk factors and treatment contexts, such as providing treatment in dental or medical offices [32]. Second, future research is needed to examine social and industrial factors that support tobacco use and evaluate occupationally tailored interventions to help firefighters to successfully quit. This is particularly important given the high proportion of dual users found in this sample. Third, fire service organizations should address and focus more on SLT cessation programs in order to reduce the risks of SLT-related illnesses given that SLT use is associated with some cancers and other oral diseases [8]. Finally, because of the exceptionally high levels of SLT and dual use in the fire service, and its unique job characteristics (e.g., spending extended periods of time living together during extended shifts, frequently working in outside weather conditions, and having high level of interdependence among crews), intervention programs should be specifically developed and tailored for this occupational group.

The current findings support earlier studies reporting that SLT products were more likely to be used by White adults than among minority respondents [9, 17]. For example, Rodu and Cole [1] found that nearly all SLT users were Caucasian. In addition, previous studies generally show SLT and other tobacco use cluster with other health risks, particularly substance use [17–19]. For instance, Lando et al., [17] found that SLT users reported more total alcohol use and binge drinking compared to never users. Alcohol use also has been associated with increased tobacco consumption including SLT [9, 17]. Although both SLT users and dual users in this sample were more likely to drink alcohol and binge drink, they were not more likely to engage in occupational risk-taking such as not wearing their SCBA during a fire. 

In contrast, there have been extensive data indicating that cigarette smokers demonstrate higher occupational risk behaviors, such as being less likely to use seat belts and more likely to use drugs and be involved in physical altercations when compared to nontobacco users [33]. Thus, SLT users and dual users are probably more health focused or have higher health consciousness than smokers. As we stated previously, detailed information on SLT and dual users' health risk behaviors can be useful for developing an SLT cessation intervention strategies that consider multiple risk factors and delivery contexts in the program design. In addition, knowing associated risk behaviors of SLT and dual can be useful for health care providers in assisting SLT and dual users quitting. 

The use of both cigarette and SLT, or dual use, was observed to be very high in this cohort. Although there were no statistically significant differences in age SLT initiation and mean numbers of SLT use between SLT users and dual users, we found that dual users started using SLT later than when they started smoking and that they used less SLT than exclusive SLT users. In addition, almost half of dual users in this sample reported using SLT after joining the fire service, indicating that fire service restrictions on smoking cigarettes might play the crucial role [11]. 

Several studies have shown that smokers who use other forms of tobacco have higher health risks compared to other tobacco users [17]. It has been suggested that dual users are trying to quit or reduce smoking by using SLT as a replacement for nicotine delivery. Also, it has been reported that dual use is associated with high levels of nicotine addiction [19], which could lead them to use SLT in addition to cigarettes. Our findings support this notion, as dual users in this sample initiated cigarette use at an earlier age than SLT use, and smoked cigarettes as much as that found in the exclusive smokers (10.00 ± 9.03; [13]). As mentioned earlier, smokers may become dual users due to smoking restrictions, health concerns, and advertising and marketing factors [11]. Therefore firefighters who use other forms of tobacco in addition to cigarettes are in particular need of research on best methods to encourage cessation and deliver treatment.

## 7. Conclusion

The prevalence rates of exclusive SLT use and dual use in this sample were 2–4 times higher than those found in national data, 13.4% versus 7.0%, and 2.6% versus 8.0%, respectively. Our findings indicated that SLT and/or dual tobacco users in this sample had higher alcohol intake and meat/snack consumption than non-tobacco users. However, SLT or dual tobacco users appear to be more health focused or had higher health consciousness than their smoker counterparts [13]. Given the exceptionally high levels of SLT and dual use in the fire service, and its unique job characteristics (such as spending extended periods of time living and working on the job third of the time, frequently working in outside weather conditions, and having high level of interdependence among crews), SLT intervention programs should be specifically developed and tailored for this occupational group. 

## Figures and Tables

**Table 1:** Participant characteristics by tobacco use (Mean ± SD, or %)^a^.

	Nontobacco users	Exclusive SLT users	Dual users
	(*n* = 285)	(*n* = 57)	(*n* = 11)
*Demographics *			
Age (years)**	39.4 ± 10.1	36.0 ± 8.3	31.2 ± 6.9
Annual family income			
≤$75,000	29.4%	39.6%	54.5%
	70.6%	60.4%	45.5%
Education			
≤College	68.9%	80.7%	63.6%
	31.1%	19.3%	36.4%
Ethnicity*			
White	87.7%	98.2%	100.0%
Minority	12.3%	1.8%	—
Marital status			
Married	79.8%	71.9%	70.0%
*Health factors *			
Weight (kg)	91.5 ± 15.2	89.8 ± 15.1	85.3 ± 9.9
BMI (kg/m^2^)	28.7 ± 4.4	28.3 ± 4.5	26.8 ± 1.9
Body fat percentages	25.5 ± 6.6	24.8 ± 6.4	22.2 ± 3.2
Waist circumference (inches)	38.4 ± 4.9	37.7 ± 4.7	36.9 ± 2.6
Having high blood pressure	58.9%	66.1%	81.8%
Maximal oxygen consumption			
Poor to very poor	22.7%	17.9%	—
Fair to superior	77.3%	82.1%	100.0%
Depression scale score	1.5 ± 1.8	2.0 ± 1.8	1.6 ± 2.1
Perceived stress scale score	10.1 ± 5.7	11.5 ± 5.6	10.3 ± 3.3
*Health-risk behaviors *			
Alcohol use in the past 30 days	81.3%	89.5%	90.9%
Binge drinking in the past 30 days***	57.9%	86.0%	90.0%
CAGE scores ≥2	10.9%	19.6%	20.0%
Driving while intoxicated in the past 30 days**	7.9%	9.8%	40.0%
Self-reported physical activity level			
Level 1-2 low to inactive	37.1%	42.1%	36.4%
Level 3–5 moderate to very high	62.9%	57.9%	63.6%
Meet minimum NFPA fitness standard	41.1%	44.6%	63.6%
Fruit/vegetable screener score			
Low (<11)	42.1%	31.6%	45.5%
Meats/snacks screener score**			
High (≥23)	52.9%	69.1%	90.9%
SCBA use during a fire	33.5%	31.6%	9.1%

^a^Compared to never users; *P < .05; **P < .01; ***P < .001.

**Table 2:** Age at initiation, and amount of SLT and cigarette use among exclusive SLT users and dual users.

	Exclusive SLT users	Dual users
	(*n* = 57)	(*n* = 11)
Age at initiation		
SLT	16.8 ± 5.1	19.1 ± 8.1
Cigarette	NA	15.4 ± 2.9
Amount used		
SLT, dips/day	4.9 ± 3.3	2.7 ± 2.9
Cigarette, cigarettes/day	NA	6.6 ± 7.4

**Table 3:** Logistic regression comparing odd ratios (OR) and 95% Confidence Interval (CI) of nontobacco users, exclusive SLT users, and dual users in relation to demographic factors and health-risk behaviors^a^.

	Exclusive SLT users	Dual users
	(*n* = 57)	(*n* = 11)
*Demographics *		
Ethnicity		
Minority	1.0	NA
White	8.3* (1.1–62.1)	NA
*Health-risk behaviors *		
Binge drinking in the past 30 days		
No	1.0	1.0
Yes	4.0** (1.7–9.3)	4.8 (0.6–39.0)
Meats/snacks screener score		
Low (<23)	1.0	1.0
High (≥23)	1.9* (1.0–3.6)	8.4* (1.1–67.2)

^a^Variables adjusted for age; *P < .05; **P < .01; ***P < .001.

## References

[1] Rodu B, Cole P (2009). Smokeless tobacco use among men in the united States, 2000 and 2005. *Journal of Oral Pathology and Medicine*.

[2] King B, Dube S, Kaufmann R, Shaw L, Pechacek T (2011). Vital signs: current cigarette smoking among adults aged ≥18 Years—United States, 2005–2010. *Morbidity and Mortality Weekly Report*.

[3] Carpenter CM, Connolly GN, Ayo-Yusuf OA, Wayne GF (2009). Developing smokeless tobacco products for smokers: an examination of tobacco industry documents. *Tobacco Control*.

[4] Bates C, Fagerström K, Jarvis MJ, Kunze M, McNeill A, Ramström L (2003). European Union policy on smokeless tobacco: a statement in favour of evidence based regulation for public health. *Tobacco Control*.

[5] Klesges RC, Sherrill-Mittleman D, Ebbert JO, Talcott GW, DeBon M (2010). Tobacco use harm reduction, elimination, and escalation in a large military cohort. *American Journal of Public Health*.

[6] Haddock CK, Weg MV, DeBon M (2001). Evidence that smokeless tobacco use is a gateway for smoking initiation in young adult males. *Preventive Medicine*.

[7] Severson HH, Forrester KK, Biglan A (2007). Use of smokeless tobacco is a risk factor for cigarette smoking. *Nicotine and Tobacco Research*.

[8] National Toxicology Program (NTP) (2011). *Report on Carcinogen*.

[9] Ebbert JO, Haddock CK, Vander Weg M, Klesges RC, Poston WSC, DeBon M (2006). Predictors of smokeless tobacco initiation in a young adult military cohort. *American Journal of Health Behavior*.

[10] Bray RM, Pemberton MR, Hourani LL *2008 Department of Defense Survey of Health Related Behaviors among Active Military Personnel: A Component of the Defense Lifestyle Assessment Program*.

[11] Poston WSC, Haddock CK, Jitnarin N, Jahnke SA (2012). A national qualitative study of tobacco use among career firefighters. *Nicotine & Tobacco Research*.

[12] Lee DJ, LeBlanc W, Fleming LE, Gómez-Marín O, Pitman T (2004). Trends in U.S. smoking-rates in occupational groups: the National Health Interview Survey 1987–1994. *Journal of Occupational and Environmental Medicine*.

[13] Haddock CK, Jitnarin N, Poston WSC, Tuley B, Jahnke SA (2011). Tobacco use among firefighters in the Central United States. *American Journal of Industrial Medicine*.

[14] Centers for Disease Control and Prevention (CDC) Smokeless Tobacco Facts. http://www.ncada-stl.org/ti/notebook/tobacco/smokeless_tobacco_facts.pdf.

[15] Substance Abuse and Mental Health Service Administration (SAMHSA) Results from the 2010 National Survey on Drug Use and Health: Summary of National Findings. *NSDUH Series H-41, HHS Publication*.

[16] Rosendahl KI, Galanti MR (2010). Predictors of early and late escalation of smokeless tobacco use and cigarette smoking among Swedish adolescents. *The Open Addiction Journal*.

[17] Lando HA, Haddock CK, Robinson LA, Klesges RC, Talcott GW (2000). Ethnic differences in patterns and correlates of age of initiation in a population of Air Force recruits. *Nicotine and Tobacco Research*.

[18] Mumford EA, Levy DT, Gitchell JG, Blackman KO (2005). Tobacco control policies and the concurrent use of smokeless tobacco and cigarettes among men, 1992–2002. *Nicotine and Tobacco Research*.

[19] Wetter DW, McClure JB, De Moor C (2002). Concomitant use of cigarettes and smokeless tobacco: prevalence, correlates, and predictors of tobacco cessation. *Preventive Medicine*.

[20] Centers for Disease Control and Prevention (CDC) Behavioral Risk Factor Surveillance System Questionnaire 2010. http://www.cdc.gov/brfss/questionnaires/pdf-ques/2010brfss.pdf.

[21] National Institutes of Health (NIH), National Heart, Lung, and Blood Institute (NHLBI) (1998). *Clinical Guidelines on the Identification, Evaluation, and Treatment of Overweight and Obesity: The Evidence Report*.

[22] Jackson AS, Blair SN, Mahar MT, Wier LT, Ross RM, Stuteville JE (1990). Prediction of functional aerobic capacity without exercise testing. *Medicine and Science in Sports and Exercise*.

[23] National Fire Protection Association (NFPA) (2006). *NFPA 1582: Standards on Comprehensive Occupational Medicine Programs for Fire Departments*.

[24] US Department of Health and Human Services (USDHHS) (2007). *Joint National Committee on Prevention, Detection, Evaluation and Treatment of High Blood Pressure*.

[25] Irwin M, Artin KH, Oxman MN (1999). Screening for depression in the older adult: criterion validity of the 10-item Center for Epidemiological Studies Depression Scale (CES-D). *Archives of Internal Medicine*.

[26] Cohen S, Williamson G, Spacapam S, Oskamp S (1988). Perceived stress in a probability sample of the U.S.. *The Social Psychology of Health: Claremont Symposium on Applied Social Psychology*.

[27] Substance Abuse and Mental Health Services Administration (SAMHSA), Office of Applied Studies *2010 National Survey on Drug Use and Health*.

[28] Vander Weg MW, DeBon M, Sherrill-Mittleman D, Klesges RC, Relyea GE (2006). Binge drinking, drinking and driving, and riding with a driver who had been drinking heavily among air national guard and air force reserve personnel. *Military Medicine*.

[29] O’Brien CP (2008). The CAGE questionnaire for detection of alcoholism: a remarkably useful but simple tool. *Journal of the American Medical Association*.

[30] Block G, Gillespie C, Rosenbaum EH, Jenson C (2000). A rapid food screener to assess fat and fruit and vegetable intake. *American Journal of Preventive Medicine*.

[31] Holiday DB, McLarty JW, Yanagihara RH, Riley L, Shepherd SB (1995). Two biochemical markers effectively used to separate smokeless tobacco users from smokers and nonusers. *Southern Medical Journal*.

[32] Severson HH (2003). What have we learned from 20 years of research on smokeless tobacco cessation?. *American Journal of the Medical Sciences*.

[33] Bray RM, Fairbank JA, Marsden ME (1999). Stress and substance use among military women and men. *American Journal of Drug and Alcohol Abuse*.

